# Deciphering functional differentiation of elements in high-entropy spinel oxides as ultralong-life anodes in lithium-ion batteries

**DOI:** 10.1039/d5sc10185a

**Published:** 2026-01-22

**Authors:** Han-Hao Liu, Jia-Lin Yang, Zhen-Yi Gu, Yue Liu, Xiao-Tong Wang, Chuan-Yu Zheng, Changshan Xu, Dai-Huo Liu, Wei Guo, Xing-Long Wu

**Affiliations:** a State Key Laboratory of Integrated Optoelectronics, MOE Key Laboratory for UV Light-Emitting Materials and Technology, School of Physics, Northeast Normal University Changchun Jilin 130024 P. R. China xinglong@nenu.edu.cn; b Department of Chemistry, Northeast Normal University Changchun Jilin 130024 P. R. China; c Key Laboratory of Organo-Pharmaceutical Chemistry of Jiangxi Province, Gannan Normal University Gan Zhou 341000 China; d Collaborative Innovation Center of Henan Province for Green Manufacturing of Fine Chemicals, School of Chemistry and Chemical Engineering, Henan Normal University Xinxiang Henan 453007 P. R. China

## Abstract

The rational design of high-entropy materials for electrochemical energy storage is hindered by an insufficient understanding of the distinct roles of constituent elements. Taking the spinel-type high-entropy oxide (CoCuMgCrFe)_3_O_4_ as a model system, this study combines density functional theory calculations with multiscale characterization to systematically reveal the functional differentiation mechanism of the constituent elements during charge/discharge processes. It is demonstrated that Cr, Co, and Fe act as “active elements” significantly enhancing the decomposition kinetics of Li_2_O, while Cu and Mg serve as “structural elements” effectively suppressing volume expansion induced by lithium intercalation, thereby improving structural stability. Critically, all high-entropy surfaces exhibit exceptionally strong adsorption of Li_2_O intermediates (adsorption energy: −5.35 to −5.64 eV), which is attributed to the synergistic modulation of the electronic structure within the high-entropy environment, thereby accelerating conversion reactions. Bond length analysis identifies the weakening of Li–O bonds near active sites, with Cr exerting the most profound influence. Furthermore, we establish the metal–oxygen bonding radius as a critical descriptor for predicting high-entropy spinel formation. This work unveils the fundamental principle of elemental cooperation in high-entropy oxides, providing crucial guidance for the targeted design of high-performance multicomponent electrodes.

## Abstract 2

This study proposes a functional screening strategy guided by a “structure–property–performance” framework for the rational design of high-entropy electrode materials.

## Graphical abstract

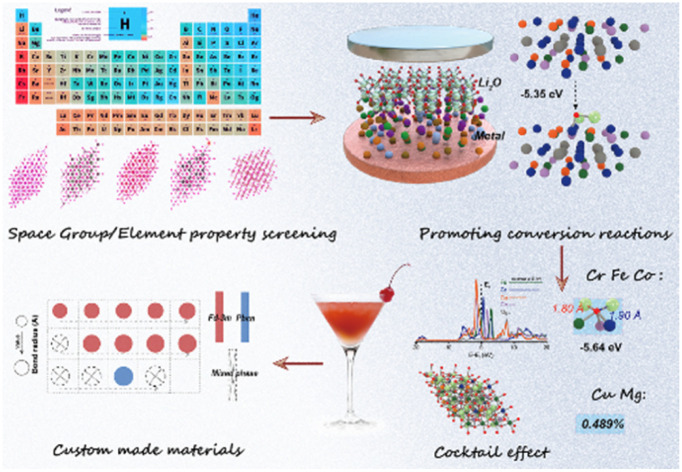

## Introduction

As a cutting-edge direction in materials science and energy storage, the high-entropy strategy offers innovative solutions to overcome performance limitations of conventional electrode materials.^1^ This approach integrates multiple metal cations synergistically, constructing multicomponent systems characterized by high configurational entropy, lattice distortion, sluggish diffusion effects, and the “cocktail effect”, significantly enhancing structural stability and tunability of physicochemical properties.^2,3^ Notably, the “cocktail effect” holds particular significance for electrode design due to its ability to strengthen conversion reaction mechanisms and its close association with elemental screening and high-entropy phase formation.^4^ High-entropy oxides (HEOs) have emerged as a new class of high-performance anode materials for lithium-ion batteries, demonstrating exceptional structural stability, cycling longevity, and rate capability owing to their unique multi-cation configuration and entropy-driven phase stabilization.^5^ While recent research has predominantly focused on performance optimization, the fundamental role of high entropy in regulating reaction pathways, stabilizing intermediate phases, and designing high-entropy phases and mechanisms remains underexplored.^6,7^ A critical gap exists in the theoretical framework correlating entropy-dominated thermodynamics with electrochemical behavior, particularly in understanding how configurational entropy modulates lithium storage mechanisms.

In particular, the structure–activity relationship between elemental functional differentiation and overall electrochemical behavior remains unsystematically established. Oxide materials, with their diverse structural types and conversion reaction activity, serve as ideal platforms for studying high-entropy systems.^8^ Previous studies indicate that introducing multicomponent active metals increases electron transfer numbers, while the “cocktail effect” suppresses dissolution of active components during cycling, further optimizing electrode durability.^9–11^ Nevertheless, two major challenges persist in achieving high-performance HEOs: first, successfully incorporating multiple transition metals into the crystal lattice while maintaining structural integrity; second, the absence of clear criteria for identifying elemental functions and guiding synthesis pathways, resulting in empirical trial-and-error approaches for composition optimization.^8,12^

To address these issues, this study focuses on spinel-type HEOs, aiming to elucidate the functional differentiation mechanisms of constituent elements during energy storage and establish corresponding rational design principles. Leveraging magnesium's known role in stabilizing spinel structures, we selected elements with similar fundamental properties and successfully synthesized the (CoCuMgCrFe)_3_O_4_ material system. Through combined density functional theory (DFT) calculations and systematic experimental validation, we revealed the evolution of electronic structures under multicomponent conditions and identified specific contributions of individual elements to conversion reaction kinetics and structural stability, providing new mechanistic insights into the “cocktail effect”. The HEO in this work demonstrates a remarkable elemental functional differentiation, where Cr, Co, and Fe serve as highly efficient “active elements” that significantly promote the decomposition of Li_2_O and enhance conversion reaction kinetics, while Cu and Mg perform as critical “structural stabilizers” that effectively suppress lattice strain and volume variation during lithiation/delithiation processes. This is evidenced by outstanding capacity retention after 10 000 cycles at a high current density of 5 A g^−1^, combined with remarkable rate capability and low charge-transfer impedance. Furthermore, by comparing electrochemical behaviors of samples with different elemental configurations, we clarified correlations between elemental combinations and performance outputs, offering both theoretical foundations and practical routes for targeted design of high-performance high-entropy electrode materials.

## Results and discussion

**Fig. 1:**
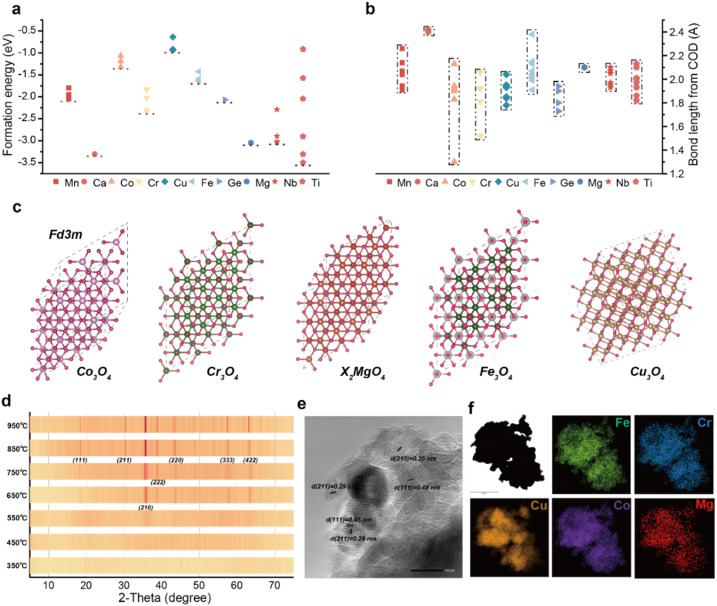
Basic properties of common elements in oxides. Statistical analysis of basic properties of oxides and model analysis: (a) formation energy for common metal elements, (b) bond length for common metal elements, and (c) characterization of high-entropy oxides. (d) XRD patterns of high-entropy oxides at different temperatures, (e) high-resolution TEM of high-entropy oxides, and (f) mapping image of high-entropy oxides.

The preparation of high-entropy oxides has long been challenged by phase separation issues arising from significant physicochemical differences among constituent elements. As pioneeringly highlighted by Rost *et al.*,^13^ phase stability in multi-component systems remains a central challenge in materials design. To systematically investigate the influence of elemental properties on the formation of high-entropy phases and mitigate the impact of elemental disparities, we first conducted a statistical analysis of the formation energies and ionic bond lengths of ten candidate elements frequently used in oxide anodes (Fig. 1a and b).^14^ Elements such as Co, Cu, Mg, Cr, and Fe were identified with low formation energies and suitable ionic bond lengths, indicating their enhanced ability to overcome phase separation and form stable oxide structures. These elements not only facilitate reduced mixing enthalpy and promote solid solution formation but also optimize electrochemical performance through a pronounced “cocktail effect”. Notably, all these elements can crystallize in the spinel structure (Fig. 1c), which adopts the *Fd*3̄*m* space group and offers high stability and favorable ion transport properties, making it an ideal platform for studying the cocktail effect and enhancing electrochemical performance.

Therefore, we selected the Co–Cu–Mg–Cr–Fe combination as the primary composition for the high-entropy oxide, aiming to leverage synergistic effects to optimize material performance and probe the role of each element. A straightforward precursor calcination method was employed to synthesize the high-entropy spinel phase (see Experimental section in SI for details). X-ray powder diffraction (Fig. S1a) confirmed the successful formation of a cubic spinel lattice (PDF-97-002-4493). High-resolution XPS spectra further verified the presence and corresponding valence states of all five elements within the spinel structure (Fig. S1b–f). Scanning electron microscopy (SEM) images (Fig. S2a and b) reveal a nanoplate-assembled morphology with an average particle size of ∼5 µm. The annealed oxide has retained this stacked morphology inherited from the layered hydroxide precursor, which enhances the exposure of electrochemically active sites. Furthermore, investigation of the phase evolution during heat treatment (Fig. 1d) reveals an entropy-driven kinetic mechanism for phase formation. XRD patterns show that after treatment at 950 °C, the sample exhibits typical single-phase spinel characteristics without detectable secondary phases, indicating the achievement of an entropy-dominated thermodynamic equilibrium. The sequential development of dominant crystal facets illustrated the growth process of the high-entropy spinel. High-resolution transmission electron microscopy (Fig. 1e) reveals clear lattice fringes with a spacing of 0.25 nm, corresponding to the (210) plane of the spinel phase, consistent with XRD results. Crucially, elemental mapping (Fig. 1f) demonstrates a homogeneous distribution of Co, Cu, Mg, Cr, and Fe, confirming the formation of a high-entropy oxide. This uniform elemental distribution is a key feature of high-entropy materials, contributing to enhanced structural stability and performance consistency.

**Fig. 2:**
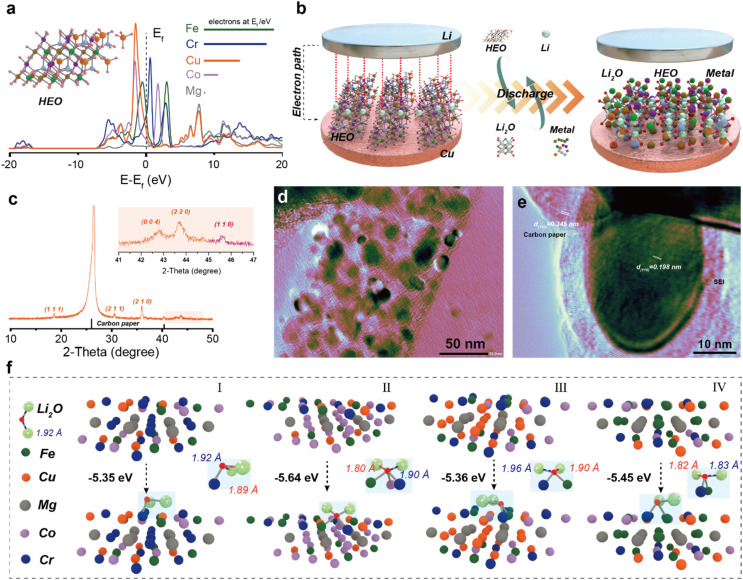
Characterization of the Li-ion energy storage process. (a) PDOS curve of high-entropy oxides (the crystal has undergone full convergence), (b) schematic diagram of the oxide conversion reaction, (c) XRD pattern of the high-entropy alloy after lithiation, (d and e) TEM images of the high-entropy oxide after lithiation, and (f) schematic diagrams for the adsorption of Li_2_O by high-entropy metals with different configurations.

The atomic-level processes, particularly how the high-entropy environment facilitates reaction kinetics, remain poorly understood. To address this, we combined theoretical and experimental approaches by constructing HEO models based on the special ‘quasi-random structure’ theory (inset in Fig. 2a and S3). Density of states (DOS) analysis (Fig. 2a) reveals that electronic states near the Fermi level are primarily contributed by Fe, Cr, and Cu, with their 3d orbitals forming a continuous distribution conducive to enhanced electronic conductivity. The performance of HEOs in lithium-ion batteries is closely associated with their unique conversion reaction mechanism.^11,15,16^ As illustrated in Fig. 2b, during discharge, the HEO undergoes a conversion reaction: HEO + 2nLi^+^ + 2ne^−^ → *n*Li_2_O + M alloy (where M denotes transition metals). The notable feature of the high-entropy effect is that it can stabilize the intermediate state of the transformation reaction and reduce the reaction energy barrier, which is positive for the storage of ions. To observe the formation of the high-entropy metal phase during conversion, we have coated the active material on carbon paper and performed electrochemical discharge. The XRD pattern (Fig. 2c) shows distinct diffraction peaks of the metallic phase after discharge, differing from the original spinel structure, with the (110) plane being dominant. This confirms the electrochemically driven formation of a high-entropy metal phase. TEM further validated this transformation: after cycling, initially dense HEO particles decomposed into nanoparticles uniformly dispersed on carbon fibers (Fig. 2d). HRTEM (Fig. 2e) reveals lattice fringes with a spacing of 0.198 nm, corresponding to the (110) plane of the metallic phase. Using DFT calculations, we have modeled Li_2_O adsorption on various active sites of the (110) surface. Metals with different configurations are still constructed using the ‘quasi-random structure’ principle (Fig. S4), and Li_2_O exhibits different binding capacities with different metals when the structure tends to be stable. The results (Fig. 2f) show strong adsorption energies ranging from −5.35 to −5.64 eV for different configurations, attributed to the complex electronic structure and multi-site synergy in the high-entropy surface. This enhanced adsorption capacity arises from the complex electronic structure and synergistic multi-site effects of the high-entropy surface. This not only benefits the decomposition of Li_2_O but also promotes the progress of the transformation reaction. Therefore, we conducted a statistical analysis of the Li–O bond lengths adsorbed on the surfaces of various configurations. The results reveal that significant variations occurred in the Li–O bond lengths near these active sites (Li–O bond length is 1.92 Å), indicating a weakening of the bonds and a tendency toward easier bond cleavage. Importantly, Cr, Co, and Fe serve as active sites with distinct influences on Li–O bonding, following the trend Cr > Fe > Co, which promotes efficient Li_2_O decomposition.^17,18^

**Fig. 3:**
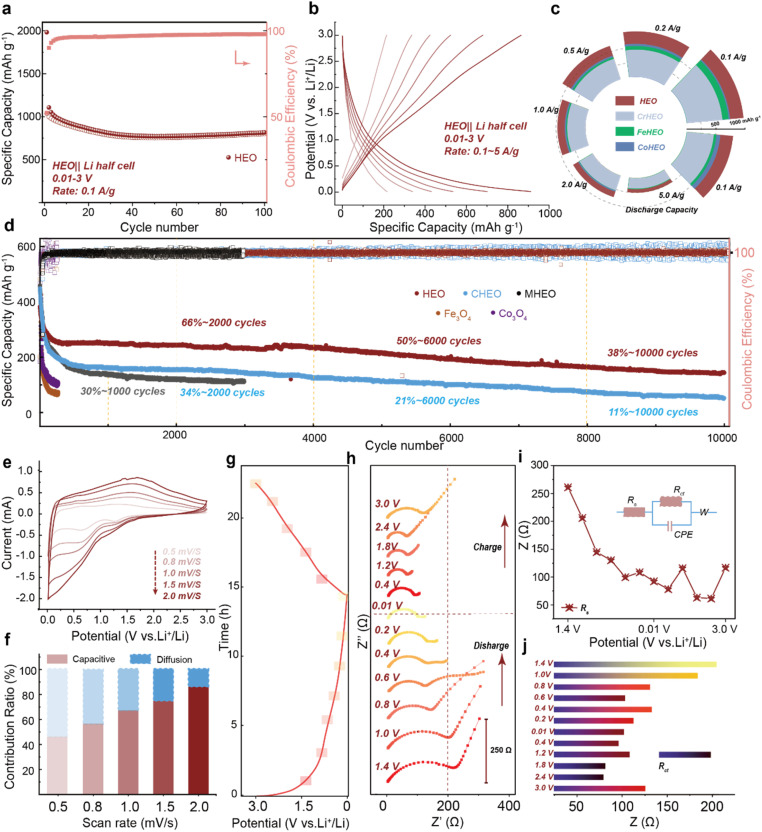
Electrochemical performance of the HEO//Li cell. (a) The current density is 0.1 A g^−1^, (b) the GCD curves of the HEO at different current densities, (c) rate performance, (d) long-term cycling stability with a current density of 5 A g^−1^, (e) CV curves at different scan rates, (f) the proportion of pseudocapacitance at different scan rates, (g) time–potential distribution curve of the HEO, (h) impedance diagrams at different potentials, and (i and j) resistance statistics chart.

Building upon the aforementioned insights to enhance the lithium storage behavior enabled by high-entropy effects, we further evaluated the electrochemical performance of the HEO anode.^19^ As shown in Fig. 3a, the material exhibits exceptional cycling stability, retaining 74% of its initial capacity after 100 cycles at 0.1 A g^−1^, outperforming conventional transition metal oxide anodes. Galvanostatic charge–discharge (GCD) curves at various current densities (Fig. 3b) highlight the unique advantages of the high-entropy system: highly reversible redox reactions with smooth voltage plateaus, in contrast to the significant hysteresis typically observed in traditional oxides.^20,21^ This behavior helps to mitigate stress accumulation and polarization during electrochemical processes. Furthermore, in the first cycle, the initial coulombic efficiency (ICE) was approximately 50%, which was due to the formation of an SEI on the electrode surface (Fig. S5). As shown in Fig. S6, the HEO//Li battery had a large irreversible peak in the first CV cycle, which corresponded to the formation of the SEI. From the second and third cycles onwards, the shape of the CV did not undergo significant changes. Under rate testing (Fig. 3c), the HEO delivered capacities of 980 mAh g^−1^ at 0.1 A g^−1^ and 250 mAh g^−1^ at 5 A g^−1^. Notably, upon returning to the initial low current rate, the capacity nearly fully recovered, demonstrating outstanding rate capability. Compared to the full-composition HEO, samples lacking Cr, Fe, or Co showed not only reduced initial capacity but also accelerated decay at high rates (*e.g.*, 5 A g^−1^), underscoring the critical role of these elements in facilitating Li_2_O decomposition (Fig. S7). This observation aligns with the trend of Li–O bond modulation (Cr > Fe > Co) identified earlier, providing further evidence for the high-entropy-enhanced conversion reaction. Following the verification of the storage capacity and rate performance of the HEO, an investigation into its long-term high-rate stability was conducted. The cocktail effect, which represents the most distinctive feature of the high-entropy concept, has consistently enabled researchers to select elements in a strategic manner to enhance overall performances.^2,16^ Based on the previous insights, Cr, Fe, and Co effectively promote the transformation reaction of high-entropy oxides in the electrochemical process by influencing Li_2_O, and we call them “active elements”. However, according to previous studies, inert elements often play a role in stabilizing the crystal structure.^6,17^

We try to reveal the role of Cu and Mg in stabilizing the crystal structure by the long-term cycling stability test results (Fig. 3d). The full-composition HEO retained 3.5 times the capacity retention of a Cu-deficient sample after 10 000 cycles at 5 A g^−1^ (Fig. S8). Meanwhile, absence of Mg led to rapid capacity decay to 30% after only 1000 cycles. Although Mg's structural role has been reported,^9^ the stabilizing effect of Cu particularly in enhancing cycling life and electronic properties represents a new insight for HEO design. In addition, the oxides of a single metal have demonstrated a rapidly decaying capacity. Kinetic analyses provided a deeper understanding: minor peak shifts in cyclic voltammetry (CV) curves under increasing scan rates (Fig. 3e) indicate highly reversible reactions. Pseudocapacitive contributions dominated (85.2% at 2.0 mV s^−1^, Fig. 3f), suggesting surface-controlled charge storage beneficial for fast ion transport. Electrochemical impedance spectroscopy (EIS) at different potentials (Fig. 3g and h) revealed low charge-transfer resistance (*R*_ct_ ≈ 200 Ω in the fresh electrode), which further decreased to 100 Ω during discharge and only slightly increased to 125 Ω upon recharge (Fig. 3i and j). The corresponding fitting circuit diagrams are shown in SI Fig. S9.^22,23^ This unique low-impedance characteristic underscores the role of high-entropy phases in facilitating interfacial charge transfer and reaction kinetics.

**Fig. 4:**
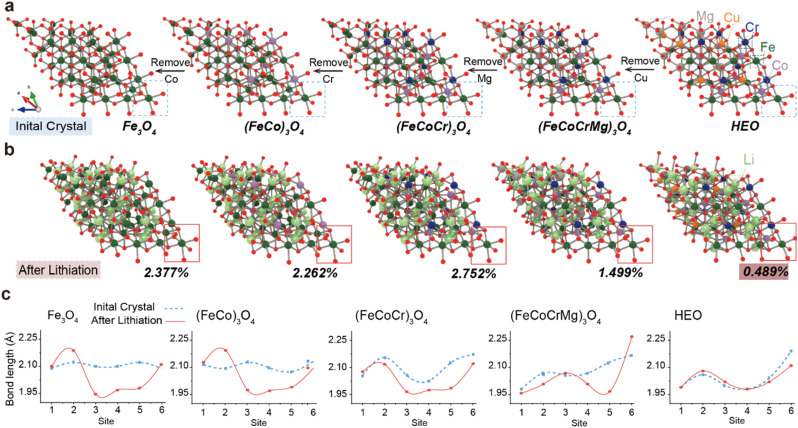
Schematic diagram of the DTF calculation for the changes in volume and chemical bonds of oxides composed of different elements before and after lithiation. The structure has undergone thorough structural optimization. (a) The oxide structure of the spinel composed of five different elements after removing the elements from right to left as shown. (b) The five spinel structures after lithiation corresponding to (a). (c) Statistics of the metal–O bond lengths at the lower right corner of five spinel structures before and after lithiation.

Subsequently, to further investigate the synergistic mechanisms of multi-element interactions in HEOs, particularly the critical role of Cu and Mg in suppressing volume expansion during lithium insertion, we constructed five spinel oxide models (Fig. 4a). By sequentially removing Cu, Mg, Cr, and Co elements, we systematically evaluated the influence of compositional variation on structural stability during electrochemical cycling. This approach effectively decouples structural contributions from purely entropic effects and confirms that the enhanced cycling stability originates from the structural stabilization by Cu and Mg, rather than configurational entropy alone. Notably, the configurational entropy decreases gradually from left to right in the model series. Quantitative analysis of volume change during lithiation (Fig. 4b) reveals that the full-composition HEO shows the smallest volume expansion (Δ*V* = 0.489) after insertion of Li atoms. Removal of Cu led to a sharp increase in volume change (Δ*V* = 1.499), while further removal of Mg resulted in even more severe expansion (Δ*V* = 2.752). These results underscore the indispensable role of Cu and Mg in mitigating lattice strain induced by Li insertion. Cu enhances bond strength and suppresses lattice relaxation due to its strong electron delocalization capability and high oxygen affinity, whereas Mg reduces cation disordering owing to its small ionic radius and stable valence state.^6,19^ This synergistic stabilization is crucial for the exceptional long-term cyclability of HEOs. Furthermore, the illustrations provide direct evidence for understanding this phenomenon from the perspective of local structure. By monitoring the bond length changes of the metal–oxygen octahedron at the edge of the spinel structure (Fig. 4c), it is found that the bond length change of the complete component HEO is the smallest after lithium intercalation, while the bond length change increased significantly with the absence of Cu and Mg. This indicates that Cu and Mg enhance the tolerance of the entire framework to the embedding of foreign ions. It is worth noting that when we remove elements with high activity (Cr, Co, and Fe), the changing trend of the volume effect is in perfect agreement with the adsorption energy calculation results mentioned earlier. Although the absence of these elements (especially Cr and Co) has a much smaller impact on volume stability than that of Cu and Mg, the direction of change is consistent with their function of promoting Li_2_O decomposition, that is, these elements mainly enhance electrochemical performance by optimizing surface reaction kinetics rather than bulk phase structure stability and enhancing electrochemical performance.

For instance, after the removal of Cr, although the crystal entropy value decreased, the volume change reduced from 2.752 to 2.262, which is contrary to the previously reported fact that the volume effect is inversely proportional to the entropy value.^24^ In contrast, this is in line with the role of Cr as an active site, but its impact intensity is far lower than the destructive effect brought about by the absence of structural stabilizing elements (Cu and Mg). This design ingeniously verifies that the contribution of Cu and Mg to the cycling stability in the electrochemical performance test mentioned earlier stems from their structural stabilizing effect rather than merely the entropy effect.

**Fig. 5:**
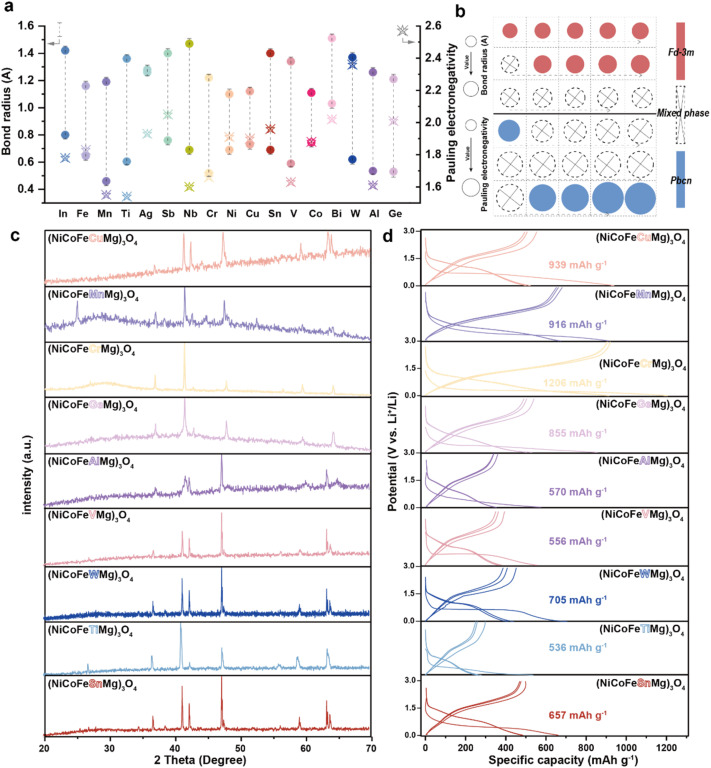
(a) Bond radius and Pauling electronegativity for common metal elements, (b) rate performance, (b) diagram of the formation mechanism of high-entropy oxides, (c) XRD patterns of high-entropy oxides, and (d) electrochemical performance of the different HEO//Li cells.

Inspired by the advantages of high configurational entropy, we further designed, synthesized, and characterized a series of HEO compounds. Using a rational element selection strategy combined with electrochemical validation, we developed multiple HEOs with tailored compositions. Fig. 5a systematically summarizes the metal–oxygen bond lengths and electronegativity values of common transition metals, providing a theoretical basis for element selection.^14^ Based on the previously identified stabilizing role of Mg, it was chosen as an essential element, followed by Ni, Co, and Fe elements with relatively short metal–oxygen bond lengths that are close to that of the Mg element and have suitable electronegativity to form a base combination. A fifth element was introduced as a variable to probe the effect of larger bond lengths. Guided by these principles, the spinel phase structure was successfully synthesized, as confirmed by XRD in Fig. 5c. Further investigation revealed the decisive influence of the metal–oxygen bond length compatibility on the formation of high-entropy phases. When elements with larger ionic radii are introduced, secondary phases emerge in the XRD patterns (SI Fig. S10) (Fig. 5b), consistent with the Hume–Rothery solid solution rules.^25^ This indicates that although high-entropy systems can somewhat extend solubility limits, atomic size difference remains a critical factor in phase stability. Interestingly, unlike reports on HEOs,^26,27^ electronegativity variation did not show a clear trend in our spinel system, suggesting crystal structure-dependent requirements for elemental properties. Notably, certain combinations formed high-entropy phases with a PBCN space group rather than the typical *Fd*3̄*m* spinel structure (SI Fig. S11). These compositions consistently contained Nb. As illustrated in Fig. 5b, the introduction of Nb significantly altered the crystallization mechanism; this offers a new perspective for designing novel high-entropy materials. In addition, Nb^5+^ is a d^0^ metal, and the empty state of its d orbital is different from that of other 3d transition metals (Fe^3+^, Co^2+^, and Cr^3+^). The Nb^5+^ of d^0^ and the 3d transition metal filled with d orbitals have significant differences in the electronic structure and coordination stability, resulting in incompatibility between the lattice and electronic structure.

The GCD curves of the successfully synthesized spinel HEO are presented in Fig. 5d. Among them, the Cr-containing spinel (Mg–Ni–Co–Fe–Cr–O) shows the best electrochemical performance. Its GCD curve shows the highest reversible capacity (1206 mAh g^−1^) at 0.1 A g^−1^ along with the smoothest discharge plateau, consistent with the previously proposed role of Cr as an active site for lithium storage. In summary, this study first screened for the synthesis of HEOs and successfully synthesized the high-entropy spinel phase. Then, through multi-scale modeling and structural analysis combined with electrochemical characterization, the functional division of different elements in high-entropy oxides was clarified. The functional screening strategy developed in this work follows a structured pathway from target space group and stability analysis, to screening based on fundamental element properties, and further to mechanistic studies that inform elemental optimization. This approach is built upon a general “structure–property–performance” design framework. It should be noted that differences in chemical reactivity (higher reactivity of sulfides) may shift the thermodynamic conditions for phase formation, potentially enabling high-entropy phases from element combinations that are unstable in oxides. Thus, the screening methodology and mechanistic paradigm established here offer a solid theoretical and methodological foundation for designing novel high-entropy electrode materials.

## Conclusions

In summary, we have demonstrated an element screening and entropy-driven synthesis strategy to successfully design and prepare a spinel-structured HEO anode material, (CoCuMgCrFe)_3_O_4_, systematically revealing the functional differentiation and synergistic mechanisms of multi-cation composition in lithium-ion battery anodes. Combining DFT calculations and multi-scale characterization, we clarified the cooperative effect of various elements in the HEO: Cr, Co, and Fe serve as “active elements” that promote the decomposition of Li_2_O and enhance conversion reaction kinetics, while Cu and Mg act as “structural elements” that significantly suppress volume expansion during lithiation and improve cycling stability. Experimental results show that this HEO exhibits excellent capacity retention after 10 000 cycles at a high current density of 5 A g^−1^, together with remarkable rate capability and low impedance. This elemental functional differentiation and synergy enable the high-entropy oxide to simultaneously achieve high capacity, long cycle life, and outstanding rate performance, surpassing conventional single-path material design strategies. Our work not only provides concrete element selection for the rational design of HEOs—the necessity to include both structural stabilizers and active elements–but also offers a new paradigm for understanding the structure–performance relationship in multi-component materials. Furthermore, through elemental substitution experiments, we validated the critical role of metal–oxygen bond lengths in the formation of high-entropy spinel phases, providing a new design principle for multi-element oxides. This study establishes a complete research framework for HEOs, from microscopic mechanisms to material design, and lays a theoretical foundation for developing next-generation high-performance energy storage materials. The design principles are expected to advance the widespread application of high-entropy functional materials in the energy sector.

## Author contributions

Han-Hao Liu, Zhen-Yi Gu and Xing-Long Wu discussed and defined the project. Han-Hao Liu initiated and led the project in experimental design, sample fabrication and procurement, and performed most of the measurements. Han-Hao Liu, Jia-Lin Yang, Yue Liu and Changshan Xu conducted the molecular simulations. Yue Liu, Jia-Lin Yang, Xiao-Tong Wang, Chuan-Yu Zheng and Han-Hao Liu participated in project discussions. Jia-Lin Yang, Yue Liu and Han-Hao Liu assisted in figure organization and technical content. Han-Hao Liu, Dai-Huo Liu, Wei Guo and Xing-Long Wu discussed and interpreted the results and prepared and finalized the manuscript.

## Conflicts of interest

There are no conflicts to declare.

## Supplementary Material

SC-OLF-D5SC10185A-s001

## Data Availability

The data supporting the findings of this study are available within the article and its supplementary information (SI). Supplementary information is available. See DOI: https://doi.org/10.1039/d5sc10185a.
