# Supplementary material for: Deciphering functional differentiation of elements in high-entropy spinel oxides as ultralong-life anodes in lithium-ion batteries
Source: Chem Sci. 2026 Jan 22;17(12):6116–24. doi: 10.1039/d5sc10185a (PMC12865591; doi:10.1039/d5sc10185a)
Supplement: SC-017-D5SC10185A-s001 [file SC-017-D5SC10185A-s001.pdf]

Supplementary Information (SI) for Chemical Science.
This journal is © The Royal Society of Chemistry 2026

Deciphering Functional Differentiation of Elements in High-Entropy Spinel Oxides as Ultralong-Life Anode in Lithium-Ion Batteries

Han-Hao Liu[a,b], Jia-Lin Yang[a], Zhen-Yi Gu*[a], Yue Liu[a], Xiao-Tong Wang[a], Chuan-Yu Zheng[c], Changshan Xu[a], Dai-Huo Liu[d], Wei Guo[c]* and Xing-Long Wu *[a]

[a] State Key Laboratory of Integrated Optoelectronics, and MOE Key Laboratory for UV Light-Emitting Materials and Technology, School of Physics, Northeast Normal University, Changchun, Jilin 130024, P. R. China.

[b] Department of Chemistry, Northeast Normal University, Changchun, Jilin 130024, P. R. China.

[c] Key Laboratory of Organo-Pharmaceutical Chemistry of Jiangxi Province, Gannan Normal University, Gan Zhou 341000, P. R. China.

[d] Collaborative Innovation Center of Henan Province for Green Manufacturing of Fine Chemicals, School of Chemistry and Chemical Engineering, Henan Normal University, Xinxiang Henan 453007, P. R. China.

*Corresponding Author(s)

Experimental Methods

Preparation of HEO: The LDH precursor was synthesized by the conventional co-precipitation method. First, dissolve 0.007 mol of nitrate (five types) in 100 mL of water to obtain A. Then, dissolve 0.2 mol of NaOH and 0.08 mol of Na_2CO_3 in 200ml of water to obtain B. Gradually add the uniform solution B to A until the PH=10. Then the mixed solution was aged for 24 hours, followed by washing with deionized water. After obtaining the powder, it was vacuum-dried at 60°C overnight. The completely dry powder was placed in a quartz crucible and then calcined in a muffle furnace at a rate of 10°C per minute to 950°C for 2 hours to obtain HEO.

Electrochemical Measurements.

The CR2032-type coin cells were assembled for electrochemical measurements. HEO was prepared by mixing active material, carbon black (Super P) and polyvinylidene fluoride (PVDF) in the mass ratio 8:1:1 with N-methyl-2-pyrrolidone on Cu foil and then drying overnight at 100 °C in a vacuum oven. A glass fibrous membrane was used as a spacer. A solution of 1M $LiPF_6$ in EC/DEC/DEC was used as the electrolyte. And the cells of both systems were tested in the ranges of 0.01-3 V (Vs Li). Cyclic voltammetry (CV) was performed on a CHI 660 electrochemical workstation and cyclic test instrument (NEWARE CT-4000). The calculation of the

pseudo-capacitor is consistent with the previous report[1]. The specific capacitance contribution ratio can be calculated according to the following formula：

$$i = k_1 v + k_2 v^{1/2} \tag{1}$$

k_1v and $k_2v^{1/2}$ represent capacitance and diffusion contribution respectively.

Computational Details

Spin-polarized density functional theory (DFT) calculations were performed using the Cambridge Serial Total Energy Package (CASTEP) code.[2] The electron exchange-correlation potential was conducted by the Perdew-Burke-Ernzerhof (PBE) functional of generalized gradient approximation (GGA)[3], and the ultrasoft pseudopotentials were employed. The kinetic energy cutoff was set to 500 eV for the plane-wave basis set. Brillouin zone integration was sampled with 3×3×1 for all samples. DFT+U method was adopted for all the simulations with U values of 2.5, 4, 2, and 2.5 for Cu, Fe, Co, and Cr, respectively. The convergence tolerances were set to be 5×10-5 eV per atom for energy, 5×10-3 Å for maximum displacement, and 0.1 eV/Å for maximum force. All of the structures were fully optimized and relaxed to the ground state.

Characterization

The crystal structure of all the samples was investigated by X-ray powder diffractometer (XRD, Bruker D8) with Cu Kα (λ = 0.15406 nm). The elemental composition and valence states of P-VPO and VPO were characterized by the X-ray photoelectron spectrum (XPS, VG Scientific with 300 W Al Kα source). The morphology features of simples were tested by scanning electron microscopy (SEM, Hitachi-SU8000) and transmission electron microscopy (TEM, JEOL-2100F).

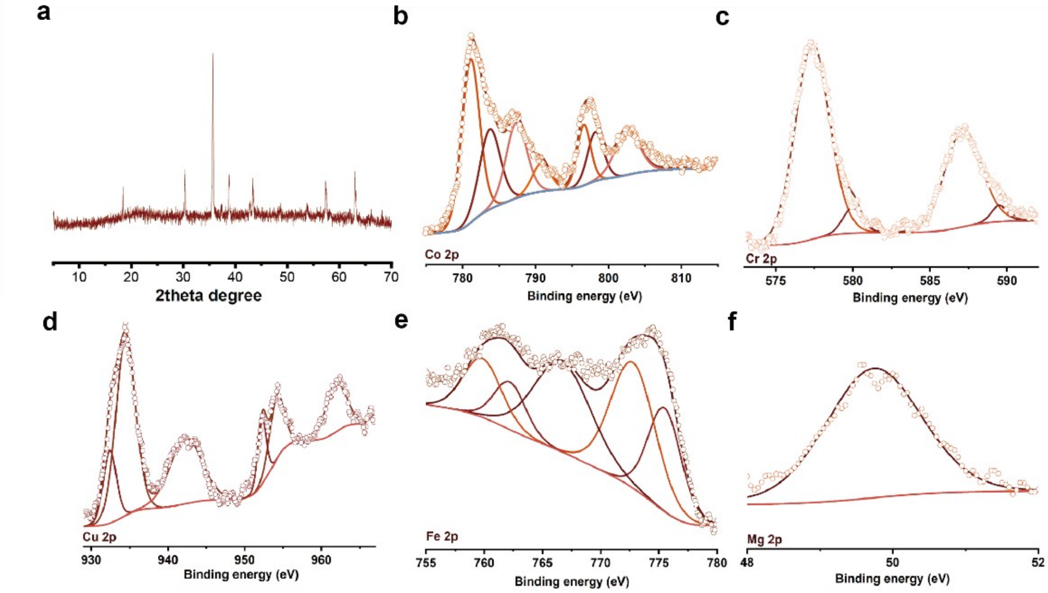

Figure S1 (a)XRD pattern of HEO, (b-f) XPS spectra of Co 2p, Cr 2p, Cu 2p, Fe 2p, Mg 2p.

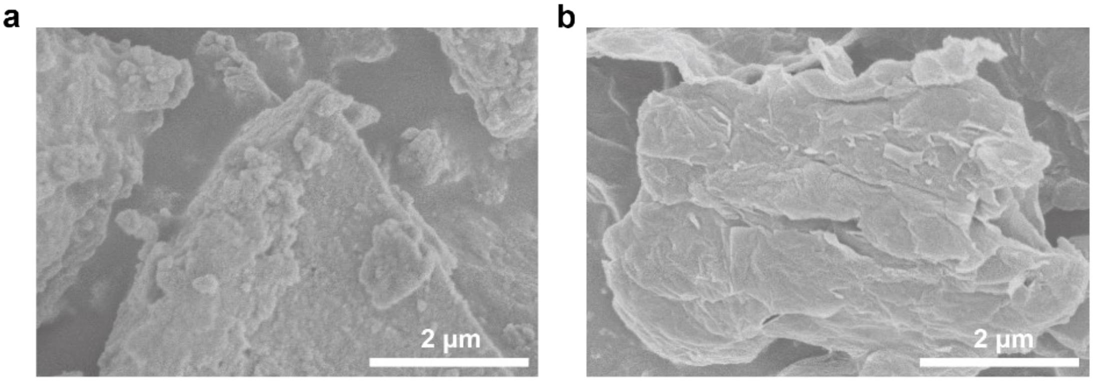

Figure S2 The SEM images of the HELDH (a), HEO (b).

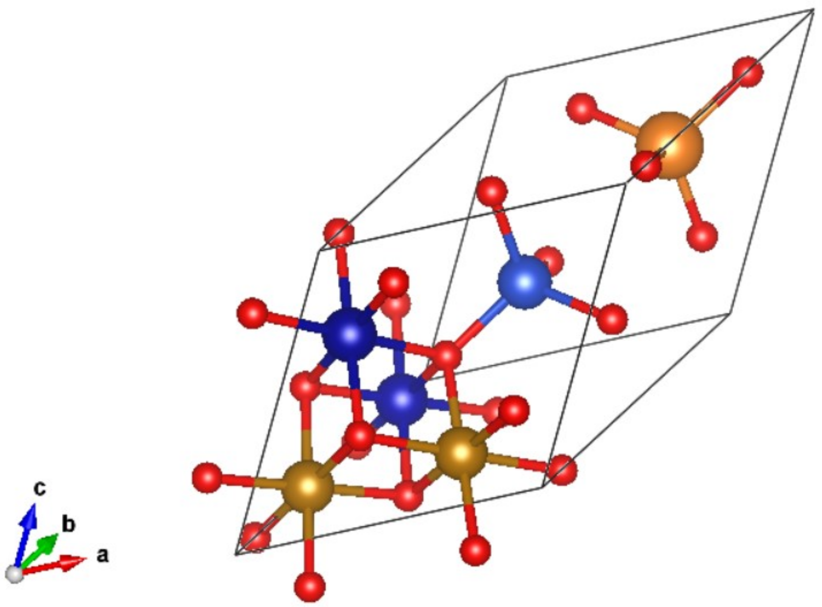

Figure S3 The optimized structure of HEO.

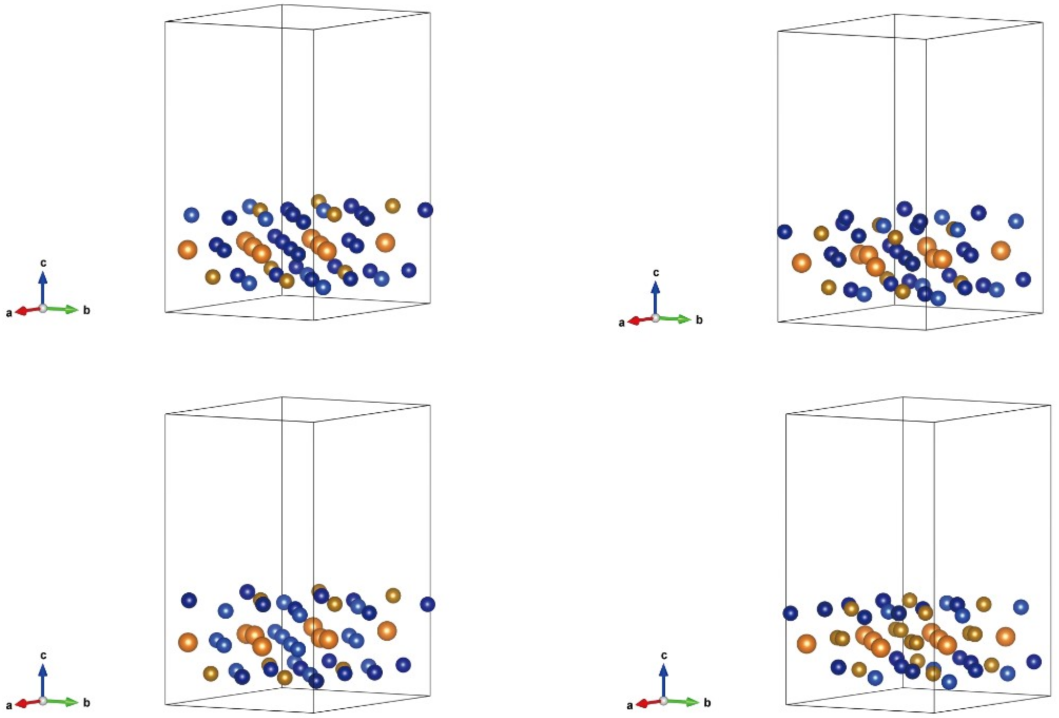

Figure S4 The optimized structure of different Alloy.

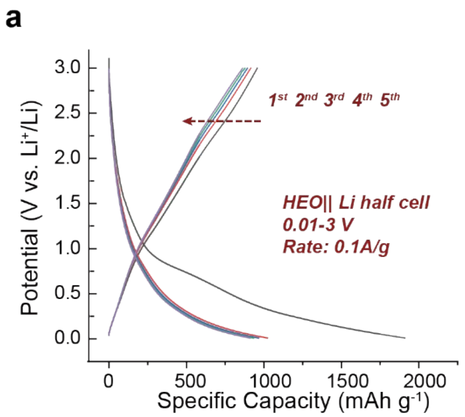

Figure S5 The GCD curves of HEO at 0.1 A/g.

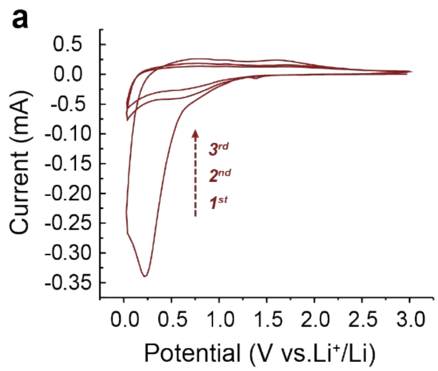

Figure S6 Cyclic voltammetry curves of HEO//Li cells at 0.5 mV/s

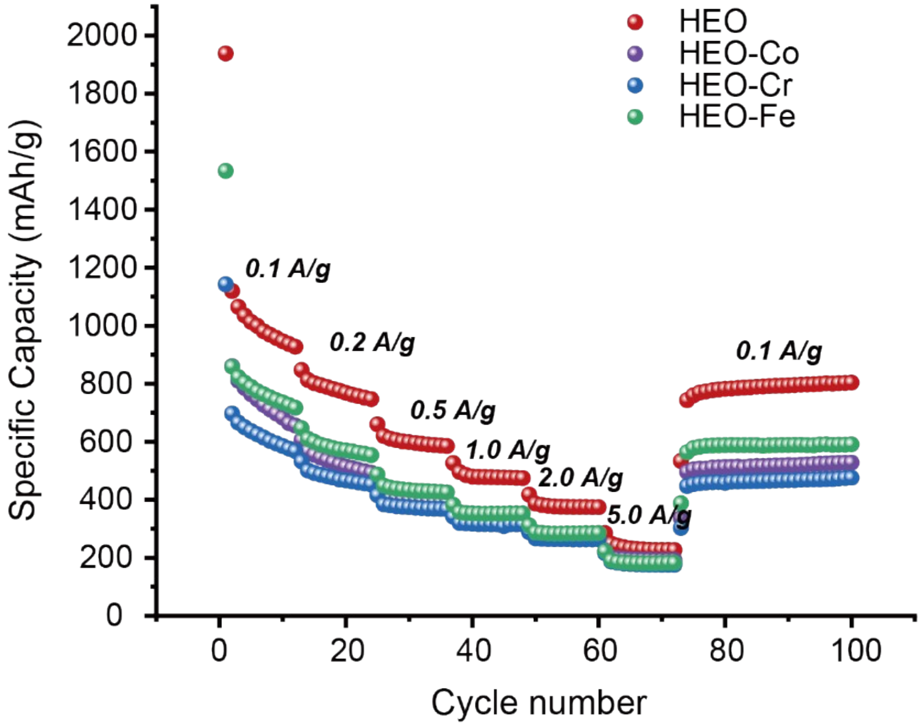

Figure S7 Rate performance of a series of HEO.

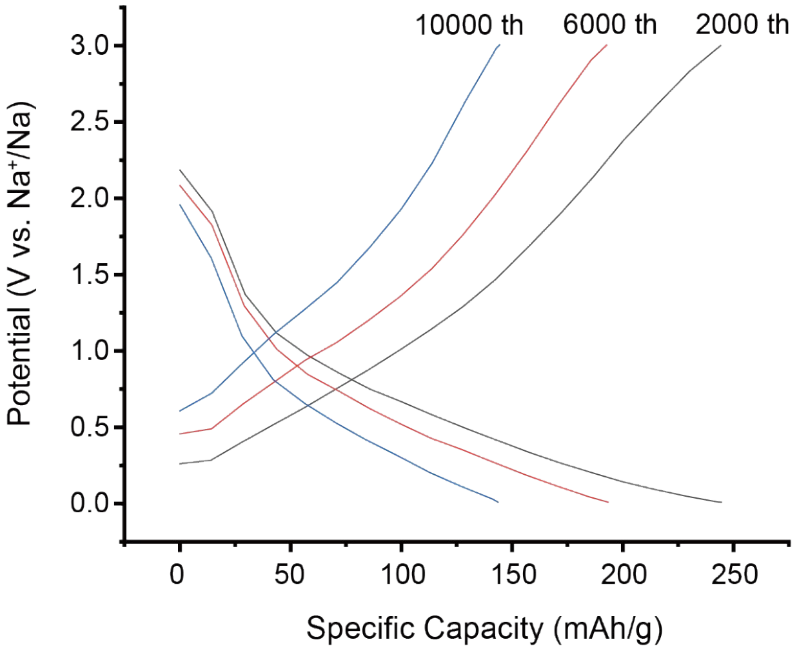

Figure S8 GCD curves of HEO at 5A g^{-1}.

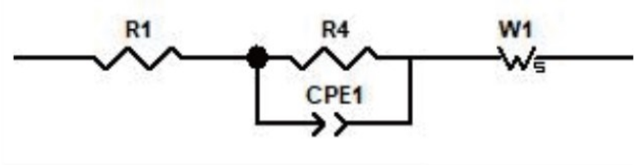

Figure S9 Fitting circuits for EIS analysis.

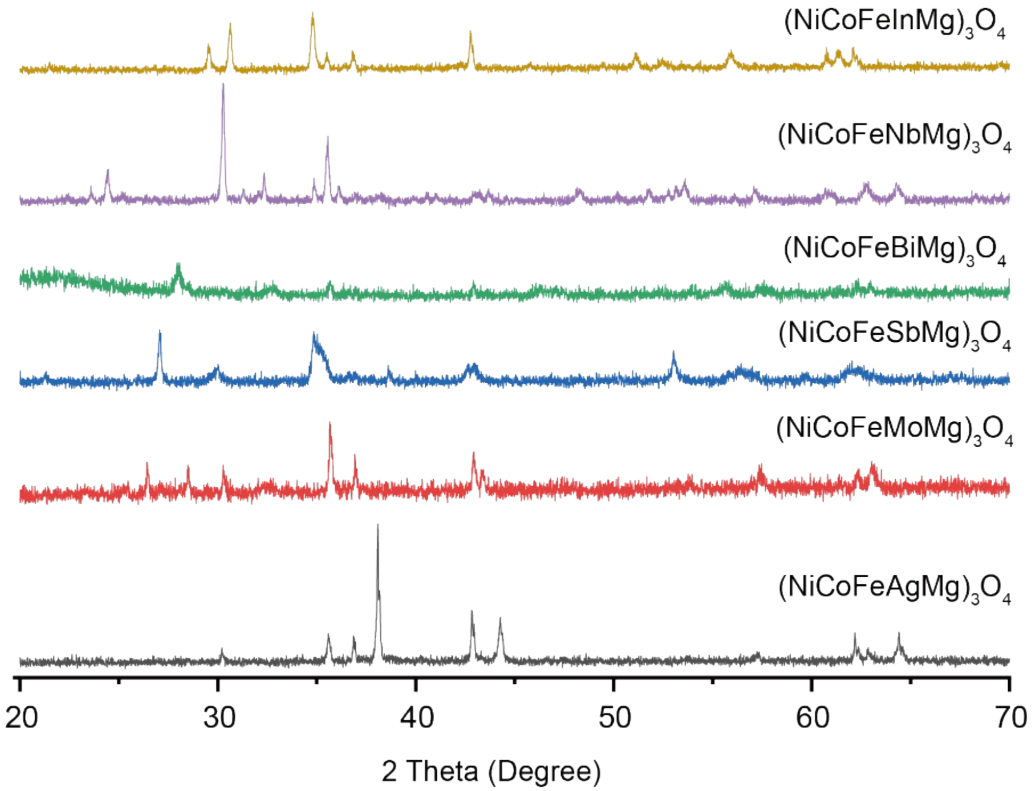

Figure S10 XRD pattern for a series of HEO.

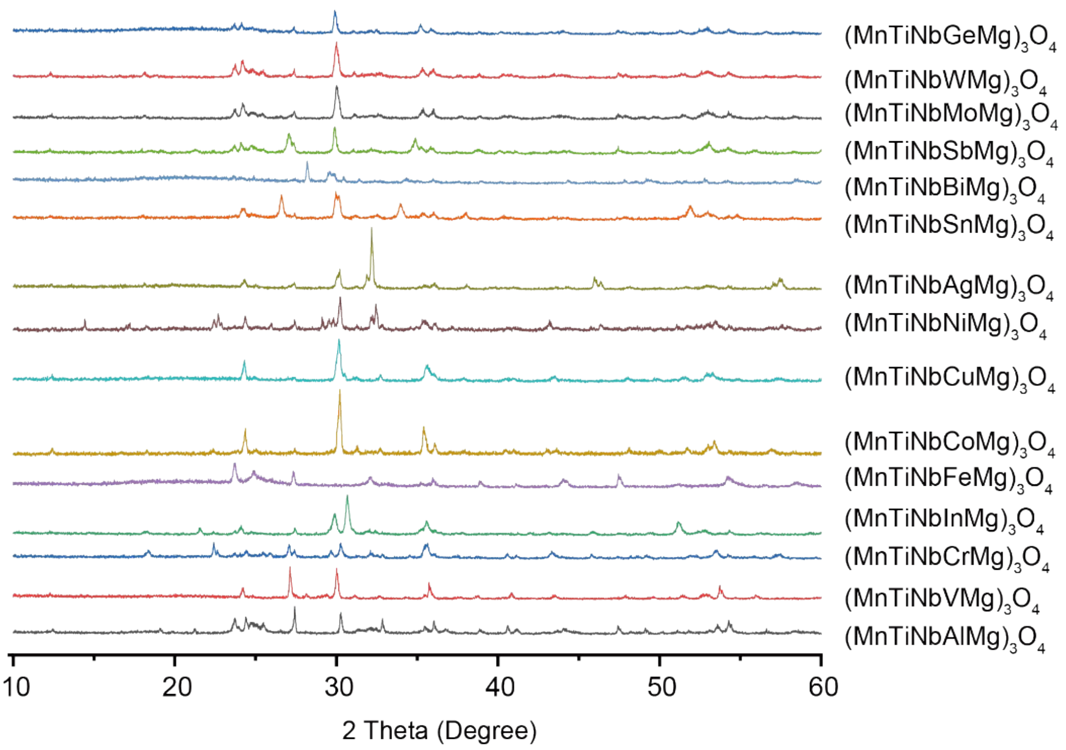

Figure S11 XRD pattern for a series of HEO.

Reference

[1] Hanhao Liu, Dan Li, Honglang Liu, Xu Wang, Yaoxin Lu, Chao Wang, Li Guo, J Colloid Interf Sci. 2023, 634, 864.

[2] S. J. Clark, M. D. Segall, C. J. Pickard, P. J. Hasnip, M. I. J. Probert, K. Refson, M. C. Payne, 2005, 220, 567.

[3] J. P. Perdew, K. Burke, M. Ernzerhof, Phys. Rev. Lett. 1996, 77, 3865.
